# 8th-grade students’ views on the concept of nanoscience through metaverse in science courses

**DOI:** 10.1038/s41598-026-51431-z

**Published:** 2026-05-02

**Authors:** Gökhan Şahin, İshak Afşin Kari̇per

**Affiliations:** Faculty of Education, Erciyes University, Kayseri, 38039 Turkey

**Keywords:** Science education, Metaverse, Nanoscience, Nanotechnology, Education, Materials science, Nanoscience and technology

## Abstract

This study aimed to examine 8th-grade students’ views on the concepts of nanotechnology and nanoscience through the use of the Metaverse in science courses. The study group sample consists of five students from both the before- and after-experience groups, all of whom are in 8th grade. This study employed a qualitative research method with a case study design. Observation, interview, and document analysis were used as data collection tools. Necessary measures have been taken to ensure the validity and reliability of the research within its scope. The data were analyzed using a content analysis approach. As a result of the interviews, data were collected and analyzed. As a result of the textual examinations, code, category, and theme were determined. The findings were presented in categories through tables, and the participants’ answers were included in direct quotations. Upon reviewing the literature, it becomes apparent that most studies in nanotechnology and nanoscience are conducted for informational purposes, typically presented as presentations or reports. Given the limited availability of nanotechnology and metaverse education, the study was divided into two groups: a before-experience group and an after-experience group. As a result of the survey, 8th-grade students experience the metaverse and have future expectations for nanotechnology and nanoscience. Their cognitive and affective interests have increased, as evidenced by their questioning why these applications cannot be applied to all courses and by their correct expression of the concepts. At the same time, it has been concluded that using rich materials to concretize abstract concepts, such as nanotechnology, facilitates their teaching. The study provides qualitative evidence that Metaverse-based instruction can enhance both cognitive and affective dimensions of science learning, offering design implications for integrating immersive technologies into middle school curricula to teach abstract concepts.

**Supplementary Information:**

The online version contains supplementary material available at 10.1038/s41598-026-51431-z.

## Introduction

Rapid advances in scientific knowledge since the early 20th century have led to widespread computer use in the development of information and communication technologies, replacing traditional methods. Change in science and technology continues, and the Metaverse is now a promising educational model for the 2020s.

So, what is the Metaverse that has recently become a favorite? There are numerous definitions and concepts related to the Metaverse, which is continually evolving in response to technological advancements^1,2^. The virtual world, which combines meta and universe, embodies the Internet^3^. The Metaverse is a term for the combination of the virtual and natural worlds^4^. The Wall Street Journal, on the other hand, described the Metaverse as a three-dimensional virtual world where avatars gather for work and other activities. The virtual world impresses us even more because it captures this reality in everything we can search and reach, such as graphics, pictures, animations, and movies^5,6^.

The Metaverse, or virtual reality, first appeared in a science fiction novel and describes interactions between natural and virtual worlds. It includes components like virtual reality (VR), gaming, entertainment, health, manufacturing, education, and social media. In augmented reality, each user sees an environment tailored to their perspective.

In 2003, a significant development occurred in the Metaverse. The “Second Life” company has created a database of a three-dimensional virtual world using augmented reality technology^7^. With advances in science and technology, the concept of the Metaverse has become a term used across almost all areas^5^. In this way, people can experience environments that may be difficult to learn in real life^8^, and it provides the opportunity to practice in high-risk working environments^9^. Following the COVID-19 pandemic, the metaverse platform has gradually gained importance, particularly for teaching abstract subjects such as nanotechnology, as distance education has become increasingly prevalent^10^.

When examining the impact of the Metaverse concept on education, it becomes more apparent that augmented reality applications offer students opportunities in education and technology, particularly in teaching abstract subjects such as nanotechnology^11^. The word “Nano” comes from the Greek word “Nanos,” which is one billionth of the criterion. The nanometer is one billionth of a meter^12^. The nanometer scale lies between the sizes of atoms and molecules^13^. Nanotechnological products are products obtained by combining atoms or molecules in diverse ways^14^. Although nanotechnology and nanoscience are used interchangeably in everyday life, they are distinct fields^15^. Nanoscience can be defined as the branch of science that tries to understand the chemical structure of nanoscale units^16^. Reviewing the literature, we find a lack of studies and training on nanotechnology^14^. Nanotechnology is included only in the 4th unit of the 12th-grade Secondary School Chemistry program^17^. According to the former national curriculum, nanotechnology was explicitly addressed only in the 4th unit of the 12th-grade Secondary School Chemistry program^17^. As per the previous curriculum, nanotechnology was only specifically integrated into the 4th unit of the 12th-grade Secondary School Chemistry course in Turkey^17^. By 2024, the Science curriculum was revised; however, the concepts of nanoscience and nanotechnology are still not explicitly and systematically integrated into students’ middle school curriculum (grades 5–8). In the revised curriculum, concepts related to the nanoscopic scale are only indirectly or implicitly linked to some topics and are not treated as an independent teaching focus. This is a clear example that abstract ideas, like nanoscience, are being delayed to higher levels of education, and that middle schools need new ways to teach.

Recent research has further underscored the urgent need to introduce nanoscience in middle school. Nanotechnology is now at the heart of medicine, electronics, materials engineering, energy storage, food science, and consumer products in everyday life, paving the way to a world in which today’s learners will inhabit and engage with a nano-enabled society. Prior research with elementary and middle school students suggests that students often face conceptual challenges with core nanoscale ideas (i.e., size and scale, the distinction between macro, micro, and nano levels, size-dependent properties) until they are grounded in explicit, developmentally appropriate instruction^18–20^. This context is also evident in existing research, as recent synthesis reports indicate that empirical studies exploring the educational effects of nanoscience are scarce at the elementary and lower-secondary levels, underscoring an ongoing need for evidence grounded in middle-school learning environments. Thus, structured nanoliteracy experiences introduced in Grade 8 offer a potential pedagogical opportunity to facilitate early conceptual development in students and to help build a solid base for the nanoscience curricula that accompany upper-secondary expectations. Recent results from a Nature-indexed study reveal that the awareness, conceptual clarity, and interest of pre-service science teachers are significantly increased following structured nanobased activities, demonstrating that nanoscience is pedagogically feasible and intellectually stimulating even for novice science students^21^. The problems that can arise when concepts related to nanotechnology are not understood have also been described in the literature^22^. Systematic reviews also suggest that delaying the introduction of nanoscience in upper secondary school limits students’ readiness for 21st-century scientific literacy and contemporary STEM professions^15,23^. Thus, the incorporation of nanoscience experiences in Year 8 is timely and necessary: it establishes basic nanoliteracy, supports modernizing curricula, reduces early misunderstandings, and enables students to make informed decisions about technologies that represent new and emergent aspects of contemporary living.

Emerging from this is evidence-based support for the view that middle-year entry into nanoscience is fundamental to orienting students for the fast-evolving, nano-enabled society of the future, rather than merely an interest or hobby. Nanotechnology now has transformative roles in medicine, electronics, materials engineering, renewable energy, and consumer products, and so to sensitize students on the fundamentals of nano-literacy, students need to have exposure to that before high school, where misconceptions concerning scale, visibility, and size-dependent properties are formed^15,24^. A new study^21^ showed that nano-based learning experiences (organized by teachers) lead to much greater levels of conceptual coherence, interest, and awareness among pre-service science teachers, teachers with longer careers, and teachers with higher levels of scientific education than Year 8 students. This suggests that when these nanoscale phenomena are well scaffolded, they are pedagogically accessible, fun, and also cognitively relevant. If such instruction is postponed until Grades 11–12, as in many national curricula, it could stifle early STEM interest and may leave children without the cognitive skills required to succeed in a scientific and technology-based world they might inhabit. For this reason, the integration of nano and nanoscience in Year 8 is both timely and integral to achieving scientific literacy and 21st-century literacy, as these concepts are embedded in our knowledge.

Conscious training of individuals in nanotechnology is crucial to both development plans and workforce formation. Given limited knowledge of nanotechnology, the development of new training, the evaluation of existing programs, and the identification of deficiencies will play a crucial role in establishing infrastructure for both teachers and students, enhancing students’ awareness, and enabling teachers to assess students’ knowledge levels^24^. In the era of interdisciplinary education, the need to employ and teach diverse methods for teaching abstract subjects, such as nanotechnology, arises^5^. The Metaverse, which provides a learning environment for both learning and living, helps teach abstract subjects such as nanotechnology. There is a notable lack of qualitative research on the role of the Metaverse in teaching abstract concepts in science education, particularly in the context of nanoscience.

The primary research question was: “What are the students’ views on the use of the Metaverse universe to make sense of the abstract nano concept and nanoscience in the science course?” The sub-problems are listed below.

- What are the students’ views on using the Metaverse universe in science lessons?
- What are the students’ views on how they found the Metaverse activity in science class?
- What are the students’ opinions about the effect of their attitudes and behaviors towards the course in the lessons held in the Metaverse universe?
- What are the students’ suggestions and opinions regarding using the Metaverse universe in education?

## Theoretical framework

This paper is grounded in constructivist learning theory, with a particular emphasis on situative learning. Constructionists believe that individuals organize their perceptions and experiences, and they interact with their environment to make sense of it^25,26^. As for abstract, scientific concepts and those learned in nanoscience and technology, they are, when introduced to middle school students for the first time, cognitively challenging due to their scale, invisibility, and complexity.

Embodied cognition is based upon the position that cognition arises to a considerable extent from sensorimotor engagement of the body with the world^27,28^. The activity in these virtual worlds, such as the Metaverse, involves multimodal transactions of ideas or experiences that allow learners to ‘act out’ or model things that are not possible in the physical world. They enable students to visualize and interact with nanoscale structures in 3D, which, in turn, helps them make more cognitive connections and develop a better understanding of complex concepts by manipulating them virtually.

Equally, situated learning theory^29^ suggests that learning is authentic when it is substantial, situated, context-embedded, and mediated by social practice. The Metaverse is the setting for students and reflects a kind of “ultra-real wet-dry-lab or physical scientific world” with which students become less observers and more players. These virtual experiences provide a learning environment where students investigate and discuss nanoscience concepts to assess their understanding.

Moreover, media richness theory^30^ supports the notion that when a medium is more interactive and engaging, it is more effective at facilitating comprehension of ambiguous and complex information. Being cloaked in visually, spatially, and experientially rich computer-mediated spaces, Metaverse provides a high-richness medium that can bridge the gap between the high complexity of abstract nanotechnology content and the cognitive preparedness of middle-school students.

By situating this work in these conceptual and theoretical frames, the inclusion of the Metaverse in middle school science is more than just technological infusion but rather a pedagogically savvy interruption in students’ conceptual grasp, attention to, and deepening of the topic at hand while engaging in ways of thinking and doing that are meaningful. These theoretical lenses serve not only as aids for contrasting but also, in theoretical discussions, to help make sense of the findings. In particular, the constructivist and situated-learning theories can account for how students reconstructed their initial conceptions of atoms and cells during their authentic, immersive practice in the Metaverse. The theory of embodied cognition may help explain why students claimed they could “see” and “touch” nanoscale entities in three-dimensional space, thereby improving their conceptual understanding. The relevance of the Metaverse to media richness theory (MRT) lies in the assumption that the Metaverse is perceived as a highly interactive medium that maintains students’ motivation and affective engagement. These relationships will be revisited in the Discussion to elucidate the findings.

## Method

**Fig. 1:**
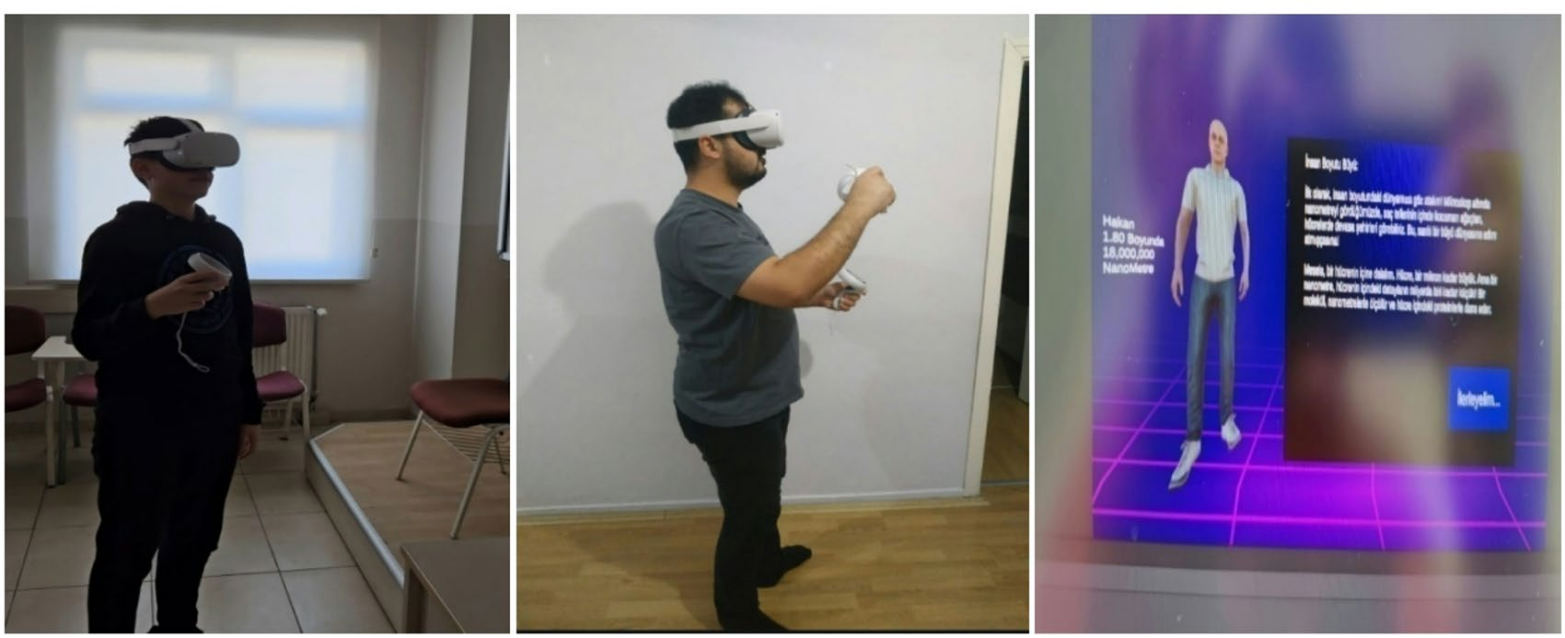
The metaverse environment features a student participating in the learning process and a teacher guiding that process; all visuals have been anonymized.

This part of the study investigates 8th-grade students’ views on the concepts of nano and nanoscience through the Metaverse during a science lesson. The details of the research model, study group, data collection, and data analysis are included. Figure 1 gives an example from the metaverse environment.

### Model of research

This research employed a case study design within a qualitative research approach. A case study can examine a phenomenon more closely within a real-life context^31^. The case examined in this research involves 8th-grade students experiencing nanoscience concepts in a metaverse-based learning environment. The case study design was chosen because this study aims to deeply investigate students’ cognitive and emotional responses in a specific learning environment. Various data collection techniques, such as interviews, observations, and document analysis, were used to enhance data diversity and robustness^32^.

### Research group

Regarding the research group, participants must meet specific criteria and be suitable for the study. This study selected a research group using a purposive sampling method. Purposive sampling allows for in-depth research by selecting information-rich situations that align with the study’s purpose^33^. The criterion sampling method was also used among the purposive sampling types. The criterion in this study is that the research group has a strong interest in the course and sufficient knowledge of internet use. The study group for this research consists of 5 female and 5 male students in 8th grade at a secondary school. In this study, purposive criterion sampling was used. This approach allows researchers to strongly select knowledgeable participants in qualitative case study research^32^. Participants were selected according to the following criteria: (1) interest in the subject and (2) proficiency in using digital tools based on pedagogical assessments of a science teacher who observed students for approximately one year. A teacher assessed students’ classroom engagement, motivation, and ability to use digital tools in a natural learning environment. Although no specific measurement tool was used, this method is consistent with context- and experiential-knowledge-based participant selection strategies used in qualitative research. All participants volunteered for the study. These criteria were not evaluated using a standard test or scale; rather, they were developed based on the science teacher’s pedagogical observations during the intervention. The teacher had taught them for approximately 1 year before participating in the study and was therefore familiar with their classroom engagement, task motivation, and use of digital and internet-based teaching and learning tools. Students who met these criteria were informed about the study, and the final study group was formed from those who volunteered to take part in the research. Such teacher-based criterion selection aligns with purposeful sampling strategies commonly used in qualitative case study research designs.

### Data collection process and tools

Phenomenological design enables direct experience^34^. To collect qualitative data, observations, interviews, and documents collected during the application process will be presented at the end of the application. In the study, observation, interviews, and document analysis were used to collect data on the concept of nanotechnology and students’ opinions on nanoscience in an 8th-grade science course through the Metaverse.

Another data collection tool we will use to evaluate the concept of nano and students’ views on nanoscience through the Metaverse in the science lesson for 8th-grade students is the interview. The interview technique involves accessing people’s experiences and attempting to understand how they make sense of them^35^. While preparing the interview questions, attention was paid to ensuring they progressed from simple to complex and were understandable. The level of the questions was reviewed by a teacher who is an expert in the field, and the questions were revised to be more comprehensible, ordered from simple to complex, and not multidimensional. Semi-structured interviewing is more advantageous for asking in-depth questions about the researched subject, for questioning, and for detailing and clarifying answers^36^. The researcher prepared the interview questions by conducting a literature review^37^. Interview questions are included in Appendix 1.

Observation, which we will use as a data collection tool, means consciously examining an object or situation^38^. The researcher’s observation was intended to understand the extent of students’ views on the metaverse learning environment and to enable students to express themselves more clearly^31^. The researcher prepared the observation questions based on the literature review^39^. Observation questions regarding the teaching of nanotechnology to 8th-grade students using Metaverse are provided in Appendix 2.

Document analysis is one of the leading methods used for qualitative research^40^. Document analysis is the acquisition of data by examining and evaluating printed and electronic materials^39^. In other words, document analysis is the examination of various written materials^36^. Document analysis complements other research methods that we will use. Document analysis was applied to 8th-grade students as a before-and-after experience to evaluate their understanding of teaching nanotechnology using the Metaverse. The science teacher ensured validity and reliability by stating that the document’s questions should be suitable for the subject’s content, understandable, and multidimensional. Document questions are given in Appendix 3.

## Data collection tools

In the study, the views of 8th-grade students on the concepts of nano and nanoscience were investigated through Metaverse during a science lesson, and observations, interviews, and document analyses were conducted. The implementation of all available data collection tools (semi-structured interview form, observation form, and document analysis questions) is detailed in the Appendices (Appendices 1–6) to enhance transparency and reproducibility.

### Validity and reliability

Validity is a concept related to how the test we apply accurately measures an individual’s feature^41^. In other words, it is the degree to which we can directly measure the property of interest without confounding other factors (Rourke & Anderson, 2004). To ensure the validity of the employee, the data obtained are presented in a detailed Table^37^.

Reliability is a test to which we apply a specific sampling method to yield comparable results across successive measurements^42^. To increase reliability, the questions were asked in the same order, and participants’ responses were conveyed in full^32^. To ensure reliability, expert opinion was sought in the preparation of semi-structured interview questions. A teacher, an expert in the field, checked the level of comprehensibility of the questions.

### Contextual description of learning environments

Both groups maintained identical nano- and nanoscience learning objectives, duration (two lesson hours), and teachers with respect to procedural equivalence. Students in the before group learned the traditional science curriculum, teacher-led explanations, textbook visuals, and short video clips, and did not have access to interactive or 3D visualization tools. After the experience, the user was immersed in a Metaverse-based learning environment designed to facilitate embodied, immersive, and sensorimotor exploration of nanoscale structures. Students manipulated 3D nanoscale objects, traveled through a virtual laboratory, and compared nano–atom–cell dimensions through spatial visualization. The different aspect was the mode of delivery, with both groups completing the same pre- and post-documentation tasks, interviews, and observation protocols.

### Description of a metaverse-based application

This study examines an application implemented in a Metaverse-based virtual learning environment to facilitate the teaching of nanoscience concepts. The application was integrated into a science lesson and completed over two class hours. The environment was a Metaverse where students interacted with 3D models of nanoscale structures such as atoms, molecules, and cells. The application platform enabled students to freely work in a virtual laboratory, manipulate objects, and observe relationships across the nano-atom-cell scales. The learning process was structured around interaction and active exploration. Students also made individual discoveries while being guided by the teacher. Activities conducted within this application included: (1) Comparing nano, atomic, and cell sizes; (2) Visualizing structures that cannot be directly observed; (3) Experiencing nanoscale environments through simulations. Unlike more traditional methods (book and video-based explanations), the Metaverse world aims to help students explain abstract scientific ideas by providing a visual, spatial, and interactive experience while studying the subject.

### Analysis of data

The qualitative data obtained were analyzed using content analysis^37^, collected through observation, interviews, and document analysis. The data analysis was performed in an iterative process of four stages: (1) coding; (2) identification of patterns and themes; (3) categorization and refining of codes/themes; and (4) interpretation.

### Coding process

The first author read and re-read all interview transcriptions, observation notes, and student papers to become familiar with the data. The set of initial codes was compiled from phrases and words resulting from students’ work. Two independent coders conducted these codings. After developing the initial coding scheme, intercoder reliability was calculated from the coded data excerpts; Cohen’s K = 0.82^43^, indicating good convergence. Discrepancies were discussed until a consensus was reached, and the codebook underwent iterative refinement to ensure accuracy. An open-source coding approach was used at the beginning of the coding process. Researchers read interview recordings and observation notes to find meaningful expressions in the data, reviewing the documents repeatedly, and then generate initial codes for these expressions. At this point, the codes were extracted directly from the data itself without a predefined code list. After comparison, the generated open-source code was clustered into similar structures and grouped into categories, with subsequent groups introduced under higher-level themes. Code was written iteratively with regular revisions and reordered as needed. For example, the expression “I understood the nanoscale better in the metaverse because it came to life in my mind” was identified with the code “understanding through visualization,” evaluated under the category “conceptual understanding,” and associated with the theme “cognitive development.” This stage was created to further increase transparency in data analysis and to clarify how the results were obtained.

### Theme development

The condensed codes were subsequently sub-grouped into sub-themes and arranged into themes. For instance, codes such as ‘fun environment,’ ‘freedom,’ ‘visual clarity,’ and ‘realistic simulation’ were all coded under “Learning Experience,” and this second-order category was merged to form a higher-order theme, “Affective Engagement.” Similarly, in the category of “Conceptual Understanding,” the codes “understanding nanoscale,” “understanding with cell/atom,” and “correct use of nanoscience terms” fell under this category.

### Presentation of findings

The themes are presented in Table 1 for transparency and clarity, and their relevance to the direct student quotes is included as evidence. For instance, a student participant mentioned that “It was easier to think what nanosize in the Metaverse was, I could see, like, find it was smaller than the atoms or cells,” exhibited the theme of “Conceptual Understanding.” “It was kind of like playing a game, but I learned some new stuff,” wrote another student; this quotation reflects the category “Affective Engagement.” The use of participants’ own words was intentional to strengthen methodological rigor^32^. This was to ensure the reliability of the analysis. The fact that the coding was systematic, that multiple coders performed the coding, a test of intercoder reliability was conducted, and direct student voice was included all contribute to the rigor of the findings.

### Ethical considerations and limitations

The study was conducted in accordance with established principles of ethical research. Before data collection, students received written consent from their parents/guardians and gave verbal assents. School authorities also approved the research activities. All subjects were presented with the same information about voluntary participation. That withdrawal could be made at any time, and all interview content would remain confidential.

The study was conducted in accordance with the best ethical practices framework for research and local guidelines. Ethical approval for the study was granted by the Erciyes University Ethics Committee (Approval No: 418, Date: 31.10.2023). Written informed consent was obtained from the parents or legal guardians of all participating students, and verbal consent was obtained from the students themselves before data collection. All research activities were also approved by the school’s administration. Participation in this study was voluntary, confidentiality was guaranteed, and participants were assured that they could withdraw from the research at any time without penalty.

### Limitations

The 8th-grade study cohort consisted of only ten students from a single public school, selected through purposive sampling. On the other hand, it restricts the extent to which the findings can be generalized. For these reasons, the findings should be understood in context and as epistemological rather than as an overarching prescription.

The sample size of this study (*n* = 10) complements the exploratory nature of data analysis commonly used in qualitative case study research. Such studies do not aim to generalize, but rather to examine experiences in detail within a specific context. Given the limited number of participants in this research, there was an opportunity to more deeply analyze students’ experiences, perceptions, and conceptual development. Therefore, this study can offer analytical rather than statistical inferences. This limitation is inherent in qualitative case study research and is not intended to produce statistically generalizable results.

This study, designed using a qualitative case study approach, is context-specific and not intended for generalization. Therefore, it cannot be directly generalized to a broader field. However, the study should be evaluated within the scope of analytical generalization. Based on these findings, conceptual inferences can be made that can be transferred to situations with similar learning environments and pedagogical characteristics. The potential impact of selection bias due to the purposive sampling method is also a significant limitation of the presentation. While it aims to increase data depth through participant involvement based on teacher observations, it also constitutes a limitation in terms of not representing diverse student profiles.

## Results

The findings have been organized thematically under three main headings in order to present a more understandable and holistic structure: (1) conceptual understanding, (2) affective interaction, and (3) learning experience.

In the research conducted on the concept of nano and their views on nanoscience through the Metaverse in the science lesson of 8th-grade students, the students in the before group (K1, K2, K3, K4, K5) and the students in the after the experience (D1, D2, D3, D4, D5) were coded. When we look at the findings obtained as a result of the preliminary test that emerged through direct expression, what is nanotechnology? After the experience, the answers given by the students in the groups before and after the experience are as follows:

### “What is nanotechnology?“Findings on the category

What is Nanotechnology? Views on the question are given in Table 1. An examination of Table 1 shows that after the Metaverse experience, students expressed the concepts of nanotechnology, nanoscale, and nanoscience in a more appropriate and scientific way. While the concepts were generally characterized by broad, vague terms in the pre-test, students demonstrated high conceptual accuracy in the post-test.

**Table 1:** Conceptual understanding: striking results (Pre–post change).

Concept	After the experience understanding (before and after the experience)	Before experience understanding (after the experience)	Striking change
Nanotechnology	Primarily defined vaguely as “a technology” or “something small”; no student defined it on a 10⁻⁹ scale	Defined accurately as “technology working at 1–100 nm scale”; used correct terminology	Correct scale knowledge appeared only after the Metaverse experience
Nanoscale	Majority: “small structure”, “do not know.”	Accurately defined as “one billionth of a meter,” compared with atoms/cells	Exact metric definition + relational reasoning gained
Nanoscience	No conceptual clarity; not recognized as a scientific branch	Described as “science studying nanoscale structures.”	Shift from confusion → correct discipline categorization
Understanding (atom–cell–nano)	“Small but not sure”; several wrong understanding	Correct hierarchical ordering: nano < atoms < cells	Students formed a scientifically accurate scale hierarchy
Showing nanoscale	“Microscope” or irrelevant tools like a meter/telescope	“Cannot be seen with regular microscopes”; requires special visualization	Conceptual shift toward scientific constraints

The results revealed that students in both the before- and after-experience groups tried to convey that nanotechnology is a science, but lacked sufficient information. These findings demonstrate that the metaverse-based learning environment enhances students’ conceptual understanding. Furthermore, it shows that it enables them to express abstract concepts within a more concrete and scientific framework.

When we examine the after-experience groups of the students in the before and after the experience groups, we see that the students in the before group stated that nanotechnology is a unit of measurement used in technological studies ranging from 1 to 100 nanometers. In contrast, the students in the group after the experience described the technology as one that emerged for use, making valuable contributions and processing small-sized objects. It was observed that the students K5 and D1 gave the same answer and expressed it as ‘a unit of measurement that is too small to be displayed under conventional microscopes.’

Where did you first hear students talk about nanotechnology, nanodimension, and nanoscience? In their responses to the question, the students reported that they generally encountered these concepts through their science teachers, videos, social media, and the books they read (listed in Appendix 1). The students’ answers to the question “What is nanosize and nanoscience?” obtained as a result of the previous groups are as follows:

### Findings in the category “What is nanoscale and nanoscience?”

What is nanosize and nanoscience? Before the group’s opinions on the question are given in Table 1. Students K1, K5, and D4 stated they were unfamiliar with nanoscale, as indicated by the previous groups, whereas students K4 and D1 described it as small structures. What is nanosize and nanoscience? After the experience, the group’s opinions on the question are given in Table 1. Students K2, K3, K4, K5, D1, D2, and D3 expressed the concept of nanosize as ' one billionth of a meter’. While students D1, D2, D4, and D5 described nanoscience as a branch of science, student K2 characterized it as a field mentioned explicitly in the context of pharmacy and medicine. Students were observed to lack knowledge of these concepts in the pre-test, but after the Metaverse experience, they were able to express them more accurately and relationally. This indicates that visualization and interaction play an important role in the learning process.

How can you show the students’ nanosize? Table 1 presents the groups’ opinions before the question. Students K1, K2, and D2 stated that they would show the nanodimension with a Microscope, while other students said they could measure their height with a telescope and a meter. Students D1 and D2 stated that they were unfamiliar with the subject. Students K1, K2, D1, D3, and D4 said that the nanoscale is tiny and difficult to demonstrate. K1 and D3 students stated that it is one billionth of a meter, while K1, K5, and D5 students described it as a minimal structure, as shown in Table 1.

Regarding the question, “If we compare with the cell, atom, and molecule you have seen in previous years, where does the nanosize fit into these concepts?” the students said they generally do not have an idea of what nanosize is, or think it is a small structure. As a result of the last test, based on the plain explanation, they stated that nanosize is a minimal concept, smaller than other concepts. After the experience, the group results of the students trained through Metaverse clearly indicated that they are smaller structures than all of these concepts (Appendix 1).

As a result of the previous groups’ responses to the question “What comes to your mind when you think of Metaverse?“, the students gave expressions such as animation, virtual reality, excitement, emotion, and freedom, and the final test result stated that they created a 3D environment for the Metaverse and presented us with an artificial environment. The final test results for the students trained through the Metaverse demonstrated that they successfully created a virtual world, simulated the environment to make it as realistic as possible, and developed a fun environment (as shown in Appendix 1). These findings indicate that students perceive the Metaverse environment not only as an enjoyable experience but also as an effective tool to support learning.

### Findings on the question “What is virtual reality?”

Table 1 provides students’ opinions before and after the experience on the question, “What is virtual reality?” Based on the previous groups’ results of students D2, D3, and D5 in the experience group, they demonstrated an understanding of virtual reality as a simulated or virtual world. In contrast, students D2, D3, D4, and D5 defined virtual reality on the axis of the artificial world, following the experience groups. Student D2, on the other hand, stated that the metaverse universe provides a free-movement environment as a result of the last test.

“Do you think the metaverse universe is permanent when teaching abstract subjects in science class?” As a result of the previous groups, the students stated that they mostly felt they were permanent and that the abstract subjects became more understandable. They noted that students who experienced the Metaverse environment reported a positive learning experience, felt the environment was conducive to learning, and did not expect to quickly forget the subject (see Appendix 1).

### Findings on the question “What is the impact of Metaverse on permanent learning?”

Table 1 gives the students’ answers before and after the experience to the question, “What is the effect of Metaverse on permanent learning?” As a result of the previous groups, D1 students reported a high visual rate. In contrast, after the experience, the results of the D4 and D5 groups indicated that the increase in the visual rate had a lasting effect on permanent learning. As a result of the previous groups, student D3 highlighted the Metaverse feature that attracted the most attention. After the experience groups, he explained that he could express it more accurately after it was sustained through continuous learning. According to the students’ statements, learning conducted in a metaverse environment is more permanent, and they also believe that visual-spatial experiences enhance the learning process.

Regarding the question “In which courses do you think the Metaverse will contribute?“, the previous groups’ results indicated that students could use it more effectively in science and mathematics lessons. In contrast, after the experience groups’ results showed that students expressed that the Metaverse environment could potentially be used in different subjects if appropriate conditions are provided (see Appendix 1).

To the question, “Where did you learn the first information about the metaverse, and what was its contribution to your life?” the students stated that they first encountered the term “metaverse” through science teachers, videos, and social media, and that the concept of the Metaverse made things easier, provided them with experience, and aided them in their school lessons (see Appendix 1). This affective engagement is consistent with media richness theory, which predicts that rich, interactive media enhance comprehension and motivation for complex content. In response to the question, “What is the place of technology in your life, and how important is it?” our students discussed the importance of technology for human beings and stated that it is now a necessity in every moment of our lives (see Appendix 1–6).

### “What do you think about words such as metaverse, nanotechnology, etc., which quickly occur in your life?” Findings on the question

What do you think about words such as Metaverse, nanotechnology, etc., which quickly take place in your life?” The answers to the question before and after the experience are given in Table 1. As a result of the experience groups, students D2, D4, and D5, who had participated in the group before, reached consensus on the concept of technology. In contrast, D1 and D3 students, who were unaware of the experience groups’ results, stated that the experience groups would enable countries to develop more quickly and that significant investments should be made in this direction.

“Throughout your educational life, at which grade level do you think Metaverse can work first? While the experience groups’ results indicate that students can be used from the 8th grade, there is not much difference after the experience groups’ results from a straight explanation. As a result of the last test, students who gained experience in the Metaverse environment stated that students’ educational level could be used at lower levels and that it would even be beneficial to use it at all levels (see Appendix 1).

When we look at the results of the observations of the 8th-grade students’ views on the concept of nano and nanoscience through the Metaverse in the science lesson, it can be concluded that the teachers who can actively carry out the process do not have difficulty in the application part, that the metaverse education, which is not economical in terms of time, attracts the attention of the students, that they look forward to the next lesson, and that the students have a different experience in the process and understand the subject better (given in Appendix 2).

When we examine the 8th-grade students’ responses to the concept of nano and nanoscience through the Metaverse in the science lesson, we can say that the students can clearly express the idea of nano and nanotechnology using Metaverse education and the magnitude it corresponds to (as shown in Appendix 3). From a situated learning perspective, students’ emphasis on permanence suggests that they experienced the activity as authentic and context-embedded, rather than a detached classroom exercise.

To further enhance the transparency and trustworthiness of the qualitative analysis, coding was systematized, and results were presented in tabular format. Codes were categorized and then combined into themes, which were illustrated by students’ verbatim quotations. These tables demonstrate the transition from raw data into actual themes. The results show that students exhibit positive cognitive and emotional development during the metaverse-based learning process. This growth is evident in conceptual accuracy, motivation, and learning retention.

**Table 2:** Conceptual understanding gains after Metaverse-based instruction.

Codes	Category	Theme	Illustrative student quote
“smaller than atoms/cells”, “one-billionth of a meter”, “microscopic structures.”	Understanding nanoscale	Conceptual Understanding	“In the Metaverse, it was easier to imagine nanosize; I could see that it was even smaller than atoms.”
“using nano for technology”, “processing tiny objects”, “scientific branch.”	Understanding nanoscience	Conceptual Understanding	“Nanoscience is how we study and use really tiny things to make technology.”
“like playing a game”, “fun environment”, “wanted to continue”, “enjoyable.”	Emotional response to VR	Affective Engagement	“It felt like a game, but I learned new things at the same time.”
“felt like a real lab”, “3D environment”, “looked real”, “free movement.”	Sense of presence	Immersion & Presence	“I felt as if I was really in a laboratory, not just watching a video.”
“helps remember”, “more permanent learning”, “will not forget easily.”	Learning durability	Perceived Learning Gains	“Because I could move and see everything, I think I may be not forget it.”
“more visual”, “high clarity”, “better than pictures/books.”	Visual richness	Media Richness Effect	“It was clearer than pictures; the 3D view made it understandable.”

**Table 3:** Affective engagement, motivation, and immersion themes emerging from Metaverse experiences.

Theme	Core evidence	Representative student quote
Motivation & Enjoyment	Students reported higher interest, curiosity, and desire to continue	“It was fun and interesting; I wanted the lesson to go on.”
Immersion & Presence	Students felt ‘inside’ a real scientific space	“I really felt like I was in a real lab, not in a classroom.”
Visual–Spatial Clarity	3D visualization made abstract concepts understandable	“Because I could see it in 3D, I finally understood how small nano really is.”
Learning persistence	Students emphasized memorability and long-term retention	“I do not think I will forget it anymore because I moved and saw it myself.”
Cognitive engagement	Students linked new concepts with prior knowledge (atom–cell–nano hierarchy)	“Now I can compare nano with atoms and cells; I can place it in my mind.”
Perceived relevance	Students reported that Metaverse should be used in other subjects as well	“Why don’t we use this in all lessons? It makes everything easier.”

Conceptual understanding gains, as well as affective engagement, motivation, and immersion themes emerging from Metaverse experiences, are presented in Tables 2 and 3, respectively. For instance, one student said, “It was easier to imagine what nanosize in the Metaverse was; I could see it as smaller than the atoms or cells.” Here, we reflect the implications of embodied cognition, where students utilized sensory-motor visualization in the Metaverse to ground their perceptions at the nanoscale relative to familiar entities, such as atoms or cells.

“It was almost like playing a game,” one student said, “but I just learned some new stuff.” This aligns with media richness theory, which suggests that interactive, multimedia environments increase learners’ motivation and engagement, particularly for complex or ambiguous content. One participant said, “I really felt as if I was in a real lab and not just watching a video,” aligning with situated learning theory, which argues that real-world, contextually rich experiences enhance the depth of engagement and perceived durability of learning. As the analysis in Tables 2 and 3 shows, the Metaverse environment not only enhances students’ conceptual understanding but also increases their motivation, interest, and learning experience in the course. (Representative student responses and sample data excerpts are presented in Appendix 6).

These tables demonstrate that coding was applied consistently and detail how students’ experiences in the Metaverse relate to conceptual understanding, affective engagement, and immersion. This aligns with embodied cognition, as students relied on sensorimotor visualization in the Metaverse to anchor abstract nanoscale concepts to familiar structures such as atoms and cells. Triangulating codes, categories, and direct quotations strengthened the credibility and trustworthiness of the findings.

## Discussion

Results of the current study suggest that Metaverse-based experiences may have a substantial positive impact on students’ conceptual understanding, affective interest, and sense of immersion in nanoscale science at the middle school level. These findings align with the primary features of constructivist learning and situated cognition, empirically supporting the theoretical perspectives described above. A significant discovery is the considerable progress in students’ ability to accurately define nanoscale concepts and to locate “nano” in relation to atoms and cells. Prior evidence suggests that young learners’ knowledge of nanoscale concepts is often inadequate or incorrect, as evidenced by their reorientation of concepts they describe^44,45^. These findings not only demonstrate a causal relationship but also reveal the learning mechanisms through which this outcome occurs. The metaverse environment enables students to experience nanoscopic structures that they cannot directly observe through three-dimensional visualization and interaction. This facilitates students’ mental concretization of abstract concepts and deepens their conceptual understanding.

In this context, the results are consistent with constructivist learning theory. Instead of passively receiving information, students interactively participated in the Metaverse environment, constructing the information themselves. Simultaneously, from a concrete cognition perspective, students’ seeing and interacting with objects in the virtual environment helped make abstract nanoscopic concepts more meaningful.

Our findings take this a step further, showing that immersive 3D visualization — in contrast to more written or video-based explanations — enables a cognitive shift from hazy understandings (“tiny things”) to scientific-scale reasoning (“one billionth of a meter”). This finding aligns with that of Mandrikas and colleagues^20^, who found that engaging in hands-on dimensional tasks improves conceptual understanding accuracy. In our findings, we discovered that through virtual embodied practice, similar or greater learning benefits are obtained with shorter instruction time. Specifically, students’ strong emotional reactions and motivation are consistent with prior research on immersive media in STEM learning. Previous studies have found that virtual reality enhances attention, engagement, enjoyment, and motivation^5,46^. This study adds empirical support to these findings. It shows that affective engagement is not just a motivational external stimulus but also a mechanism, and, in fact, an additional way of thinking beyond simple associations that leads to deeper cognitive processing of a concept. The feeling of being in an actual nano-lab that pupils observed is consistent with media richness theory^30^. It supports the hypothesis that the content of rich interactive media reduces cognitive barriers to complex science messages.

Furthermore, students indicated that the experience was memorable on a developmental level, drawing on situated learning theory^29^. Experiences with science concepts in an authentic, action-oriented context enable learners to build lasting cognitive systems. This finding aligns with Lin et al.^19^, who reported better retention under the conditions of nanotechnology camps. Our work demonstrates that the same level of permanence can be achieved without specialized physical infrastructure, but rather through a robust Metaverse environment. In this study, the authors expand the literature by providing evidence suggesting that Metaverse-based learning can overcome longstanding barriers to teaching abstract nanoscale subjects. While previous research has examined video and print, as well as short workshop instruction^14,47^, we found that immersive, non-passive immersion is instrumental in supporting middle-schoolers in making sense of phenomena at the nanoscale. By doing so, it provides an alternative to mainstream explanatory methodologies and casts the Metaverse as an educational tool rather than a technological innovation. These findings further reinforce recent arguments that introducing nanoscale concepts earlier—during the lower secondary years—is pedagogically appropriate and necessary for developing nano-literacy^15,21^.

One of the main reasons why metaverse-based learning works is that students truly experience information rather than simply learning it. Presenting a visual, spatial, and interactive element in the classroom can increase student attention and encourage active participation in learning. This is part of what makes learning more lasting and more permanent. However, our findings cannot be explained solely by the metaverse environment. Factors such as student motivation levels, novelty, and teacher guidance may also have played a role in the learning process. Therefore, these variables should be considered when evaluating the metaverse environment.

However, the findings of our study are based on a limited sample and a short-term application. Therefore, it cannot be definitively stated that the Metaverse environment will be effective in all courses or will provide long-term learning outcomes. The results provide evidence of the promise of the Metaverse for learning and could benefit from a larger sample and longer-term research to determine whether Metaverse-based learning translates its impact across a wide range of courses and into long-term learning.

### Limitations

This study has some limitations that should be noted. First, the sample was small, consisting of only 10 8th-grade students from one public school, and therefore, the findings would not be generalizable. Second, the research context—short-term implementation in a single science unit—may be insufficient to reflect the long-term impact of Metaverse-based instruction on science learning. Third, the research primarily employs qualitative approaches (interviews, observations, and document analysis), which yield detailed observational data but have inherent limitations for generalizing to broader populations. These and other limitations impose significant methodological and contextual constraints on the present findings, which should be considered exploratory and context-specific. However, they indeed constitute a reasonable basis for further and larger studies.

Furthermore, to improve methodological clarity and transparency, data collection tools are detailed in the Appendices (in Appendix 1–6). Despite the magnitude of the low speeds, this method increases the diversity in the dataset and also allows for the presentation of results that enhance triangulation.

#### Future research directions

Although this study’s findings provide an initial understanding of how Metaverse-based instructions can promote middle school students’ learning in nanoscience, further research is needed to extend and refine this insight. To enhance generalization, future work may consider a larger, more diverse sample of students spanning multiple schools and grade levels. Longitudinal studies inform whether the cognitive and affective benefits documented here are maintained. Additionally, quantitative or mixed-methods approaches could supplement qualitative findings by systematically tracking gains. Finally, future studies should examine how different Metaverse design structures (levels of interactivity, collaboration features, and gamification) influence students’ understanding, motivation, and sense of presence in virtual environments. The study can be applied in other fields to investigate the effect of the Metaverse on the teaching of abstract subjects.

## Conclusion

This study suggests how Metaverse learning environments can support the cognitive and affective dimensions of science learning related to abstract concepts, such as nanoscience. Metaverse participants tended to display more coherent and accurate models of nanotechnology and nanoscale structures and demonstrated significant associations with their existing awareness. Their sense of involvement, drive, and persistent learning suggests that the Metaverse may serve as a learning context where intellectual and emotional challenges are real. Instead of repeating its findings, the conclusion highlights the three primary contributions of the study:

The experiential 3D visualization may help young children understand the dimensions (e.g., scale) associated with nanoscience, overcoming sensory barriers. Sensory engagement and presence are not mutually exclusive, but are also key dimensions, the processes by which students engage in experiential learning and make sense of more complex information. The metaverse may offer a complementary alternative to expensive lab- and camp-based nanoscience experiences, scaling quickly to enable high-quality science learning to be incorporated into everyday classrooms. These results suggest potential opportunities for immersive virtual worlds in the future of STEM teaching and learning, particularly for teaching phenomena that cannot be observed or manipulated. Future studies should include more subjects, examine methods in parallel across different methodologies, and investigate how various characteristics of the Metaverse (such as interactivity level, collaboration tools, and gamification, etc.) affect learning at the time-based level.

## Supplementary Information

Below is the link to the electronic supplementary material.

Supplementary Material 1

Supplementary Material 2

## Data Availability

* All data generated or analyzed during this study are included in this published article.

## References

[1] William, B. III. Everything you know about the Metaverse is wrong. (2018).

[2] Lim, W. Y. B. et al. Realizing the metaverse with edge intelligence: a match made in heaven. Preprint at https://arxiv.org/abs/2201.01634 (2022).

[3] Kyle, C. Facebook wants us to live in the metaverse. (2021).

[4] Park, S. M. & Kim, Y. G. A metaverse: taxonomy, components, applications, and open challenges. *IEEE Access.* **10**, 4209–4251. 10.1109/ACCESS.2021.3140175 (2022).

[5] Demirbağ, İ. Three-dimensional virtual worlds. *J. Open. Educ. Appl. Res.* **6**, 97–112 (2020).

[6] Ayiter, E. IEEE Further dimensions: text, typography, and play in the metaverse. In Proc. Int. Conf. Cyberworlds. 10.1109/CW.2012.50 (2012).

[7] Çelik, R. Metaverse nedir? Kavramsal değerlendirme ve genel bakış. Balkan ve Yakın Doğu Sos. *Bilim Derg*. **8**, 67–74 (2022).

[8] Bakioğlu, A. & Şentuna, T. Duties of teachers and school administrators in education with the internet. *Pamukkale Univ. J. Educ. Fac.* **1**, 10–18 (2001).

[9] Altunal, I. The use of the metaverse world as an educational model and its reflections on accounting education. *J. Selcuk Univ. Vocat. Sch. Soc. Sci.* **25**, 433–443 (2022).

[10] Lee, H., Woo, D. & Yu, S. Virtual reality metaverse system supplementing remote education methods: based on aircraft maintenance simulation. *Appl. Sci.* **12**, 52667. 10.3390/app12052667 (2022).

[11] Alkan, S. & Bolat, Y. Metaverse in education: an informative literature review. *J. Int. Educ. Sci.* **9**, 267–295 (2022).

[12] Uldrich, J. & Newberry, D. *The next big thing is actually very small* (Ledo Publishing, 2005).

[13] Erkoç, Ş. *Nanoscience and nanotechnology* 2nd edn (METU, 2007).

[14] Karataş, F. Ö. & Ülker, N. Chemistry students’ level of knowledge about nanoscience and nanotechnology. *Turk. J. Sci. Educ.* **11**, 103–118 (2014).

[15] Ng, W. Nanoscience and nanotechnology for the middle years. *Teach. Sci.* **55**, 16–24 (2009).

[16] The Royal Society & The Royal Academy of Engineering. Nanoscience and nanotechnology: opportunities and uncertainties. (2004). http://www.nanotec.org.uk/finalReport.htm

[17] Ministry of National Education (MEB). Science curriculum (primary and secondary education grades 3–8). (2018).

[18] Hingant, B. & Albe, V. Nanosciences and nanotechnologies learning and teaching in secondary education: a review of literature. *Stud. Sci. Educ.* **46**, 121–152 (2010).

[19] Lin, S. Y., Wu, M. T., Cho, Y. I. & Chen, H. H. The effectiveness of a popular science promotion program on nanotechnology for elementary school students. *Res. Sci. Technol. Educ.* **33**, 22–37. 10.1080/02635143.2014.971733 (2015).

[20] Mandrikas, A., Michailidi, E. & Stavrou, D. Teaching nanotechnology in primary education. *Res. Sci. Technol. Educ.* **38**, 377–395 (2020).

[21] Tenekecigil, E. & Kariper, İ. A. Determination of the opinions of science teacher candidates about nanoscience and nanotechnology and their development through activities. *Sci. Rep.* **15**, 12458. 10.1038/s41598-025-12458-w (2025). 40691228 10.1038/s41598-025-12458-w PMC12279953

[22] Tenekecigil, E. & Kariper, İ. A. A new concept: nanophobia. *Discov Psychol.* **5**10.1007/s44202-025-00503-8 (2025).

[23] Gocmen, A., Karabulut, H. & Kariper, İ. A. Review of studies conducted in nanotechnology education: a meta-synthesis study. *Discov Educ.* **3** 169 10.1007/s44217-024-00277-6 (2024).

[24] Yawson, R. M. An epistemological framework for nanoscience and nanotechnology literacy. *Int. J. Technol. Des. Educ.* **22**, 297–310 (2012).

[25] Piaget, J. *The origins of intelligence in children* (International Universities, 1952).

[26] Vygotsky, L. S. *Mind in society: the development of higher psychological processes* (Harvard Univ. Press, 1978).

[27] Wilson, M. Six views of embodied cognition. *Psychon Bull. Rev.* **9**, 625–636. 10.3758/BF03196322 (2002). 12613670 10.3758/bf03196322

[28] Barsalou, L. W. Grounded cognition. *Annu. Rev. Psychol.* **59**, 617–645. 10.1146/annurev.psych.59.103006.093639 (2008). 17705682 10.1146/annurev.psych.59.103006.093639

[29] Lave, J. & Wenger, E. *Situated learning: legitimate peripheral participation* (Cambridge Univ. Press, 1991).

[30] Daft, R. L. & Lengel, R. H. Organizational information requirements, media richness, and structural design. *Manage. Sci.* **32**, 554–571. 10.1287/mnsc.32.5.554 (1986).

[31] Yin, R. K. *Case study research: design and methods. *5 (Sage, 2009).

[32] Creswell, J. W. & Poth, C. *N. Qualitative inquiry and research design: choosing among five approaches 4th edn* (Sage, 2018).

[33] Marshall, C. & Rossman, G. *B. Designing qualitative research* (Sage, 2014).

[34] Williams, C. Research methods. *J. Bus. Econ. Res.* **5**, 65–71 (2007).

[35] Berg, B. L. & Lune, H. *Qualitative research methods in social sciences* (Education Publishing House, 2015).

[36] Çepni, S. Introduction to research and project work 4th edn (2009).

[37] Yıldırım, A. & Şimşek, H. Qualitative research methods in social sciences (Elite, 2016).

[38] Creswell, J. W. *Qualitative inquiry and research: choosing among five traditions* (Sage, 2007).

[39] Fabric, A. The use of technology as an active learning tool in the context of hybrid education in science courses. *Trakya J. Educ.* **13**, 943–961 (2023).

[40] Argan, M. & Dinç, N. Take me to other realms! User-based metaverse event experience. *Event. Manage.* **13**, 1–15 (2022).

[41] Merriam, S. B. *Qualitative research and case study applications in education* (Jossey-Bass, 1998).

[42] Merriam, S. B. & Tisdell, E. J. Designing your study and selecting a sample. In Qualitative research: a guide to design and implementation (Wiley, 2016).

[43] Miles, M. B. & Huberman, A. *M. Qualitative data analysis: an expanded sourcebook* (Sage, 1994).

[44] Ekli, E. Investigation of primary school students’ basic knowledge and opinions about nanotechnology. M.Sc. thesis, Muğla Univ. (2010).

[45] Smith, D. *The little book of big ideas: 150 concepts and breakthroughs that transformed history* (Michael O’Mara Books, 2017).

[46] Farjon, D., Smits, A. & Voogt, J. Technology integration of pre-service teachers explained by attitudes and beliefs. *Comput. Educ.* **130**, 81–93 (2019).

[47] Harman, G. & Şeker, R. Pre-service science teachers’ awareness of the concept of nanotechnology. *J. Bingöl Univ. Inst. Soc. Sci.* **8**, 429–450 (2018).

