# Supplementary material for: 8th-grade students’ views on the concept of nanoscience through metaverse in science courses
Source: Sci Rep. 2026 May 2;16:20300. doi: 10.1038/s41598-026-51431-z (PMC13324754; doi:10.1038/s41598-026-51431-z)
Supplement: Supplementary file 1 — Supplementary Material 1 [file 41598_2026_51431_MOESM1_ESM.pdf]

Certificate of Proofreading

This document certifies that the manuscript detailed below was edited and proofread for correct English grammar, spelling, punctuation, and vocabulary at the Proofreading & Editing Office.

‘’ 8TH-GRADE STUDENTS' VIEWS ON THE CONCEPT OF NANOSCIENCE THROUGH METAVERSE IN SCIENCE COURSES’’

By

Gökhan Şahin, İshak Afşin KARİPER

December 1, 2025

English Editor
Prof. Dr. Eugene Steele

English Editor
Lecturer Ayşegül Özaslan, M.A.

English Editor
Lecturer Aslı Öztürk

Proofreading & Editing Office
Office of the Dean for Research
Erciyes University

Certificate Number: 2025.667

E-mail: editingoffice@erciyes.edu.tr

Contact: +90-352-207-66-66 (Ext: 12508)
