# Supplementary material for: 8th-grade students’ views on the concept of nanoscience through metaverse in science courses
Source: Sci Rep. 2026 May 2;16:20300. doi: 10.1038/s41598-026-51431-z (PMC13324754; doi:10.1038/s41598-026-51431-z)
Supplement: Supplementary file 2 — Supplementary Material 2 [file 41598_2026_51431_MOESM2_ESM.docx]

Appendix

**Appendix-1: 8th grade students' views on the concept of nano and nanoscience through Metaverse in science course**

Dear students, I am conducting research on their awareness of nanotechnology teaching with the Metaverse in this research. The data obtained within the scope of this research will only be used in this research, and your information will be kept strictly confidential. Thank you in advance for your contribution to the research.

Interview questions:

1-Your Gender : *( )Girl, ( )Boy*

2. What is nanotechnology?

3. Where did you first hear the terms nanotechnology, nanodimension, nanoscience?

4.What is nanosize and nanoscience?

5. If you want to show the nano size, how can you show it?

6. If we compare the cell, atom, and molecule we have seen in previous years, where does the nano dimension fit into these concepts?

7. What comes to mind when you think of the Metaverse?

8. Have you received any training on the Metaverse universe before? If so, can you share your experiences with us?

9. What is virtual reality?

10. Have you had a chance to try VR glasses? If so, can you share how you feel?

11. Do you think the metaverse universe is permanent in teaching abstract subjects in science lessons? If yes, what is the reason that led you to this thought?

12. What do you think is the effect of Metaverse on permanent learning?

13. In which courses do you think the Metaverse will contribute?

14. Where did you learn the first information about Metaverse, and what was its contribution to your life?

15. What is the place of technology in your life, and how important is it?

16. What do you think about words such as Metaverse, nanotechnology, etc., quickly occurring in your life?

17. Which grade level do you think the Metaverse can work for the first time during your education?

Appendix-2: **8th-grade students' observation questions on the concept of nano and nanoscience through the Metaverse in science lesson:**

1. Did the students know about nano, nanotechnology, and nanoscience?

YES ( ) NO ( )

2. Were the students able to relate the nanodimension to their knowledge from previous years?

YES ( ) NO ( )

3-Were the students impressed when they heard about the metaverse environment?

YES ( ) NO ( )

4-Are the students looking forward to wearing the VR glasses?

YES ( ) NO ( )

5 Were the students happy during the process?

YES ( ) NO ( )

6-Did the students feel that they had a different experience?

YES ( ) NO ( )

7-Do the students learn the subject better in the metaverse environment?

YES ( ) NO ( )

8-Do you believe that the Metaverse environment can be used in other courses?

YES ( ) NO ( )

9-Have the students looked forward to the next lesson?

YES ( ) NO ( )

10-Did the teaching method affect learning?

YES ( ) NO ( )

11- Is the use of Metaverse economical in terms of time?

YES ( ) NO ( )

12-Do you think that the teacher had difficulty in the process?

YES ( ) NO ( )

13-Does the Metaverse environment reduce the burden of teaching?

YES ( ) NO ( )

14-Does the effective use of the Metaverse environment increase the teacher's motivation?

YES ( ) NO ( )

Appendix-3: 8th**-grade students' views on the concept of nano and nanoscience through the Metaverse in science course document questions:**

1 How can you express nanotechnology, an abstract subject, by drawing it visually?

2-How do you draw the nano dimension as a visual?

How can you show nano size within 3-1 meter?

4-What visual structure comes to mind when you think of nanotechnology?

5-What kind of essay would you write about teaching nanotechnology with Metaverse?

Appendix 4: **Photographs of 8th Grade Students Used in the Examination of Students' Views on the Concept of Nano and Nanoscience Through Metaverse.**


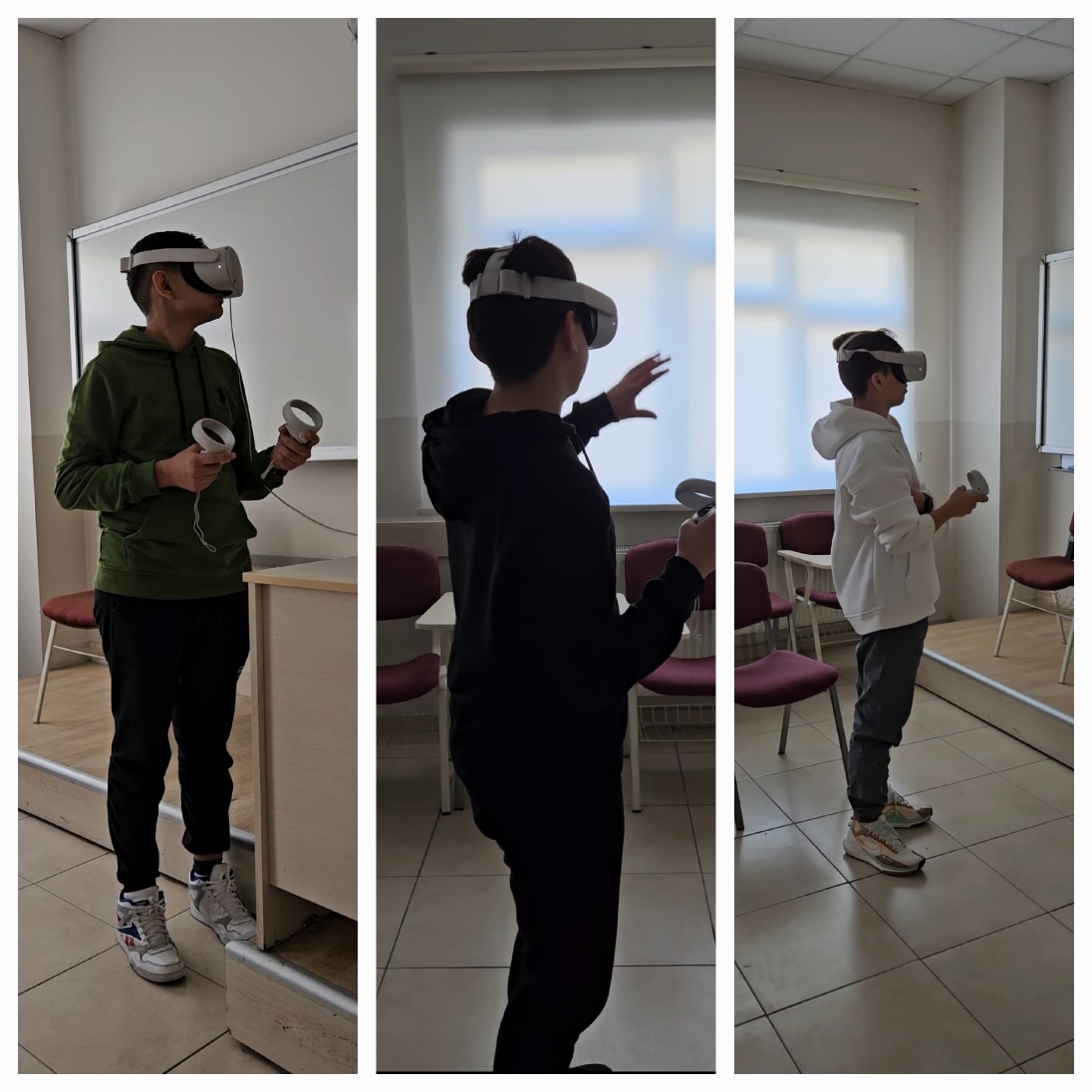


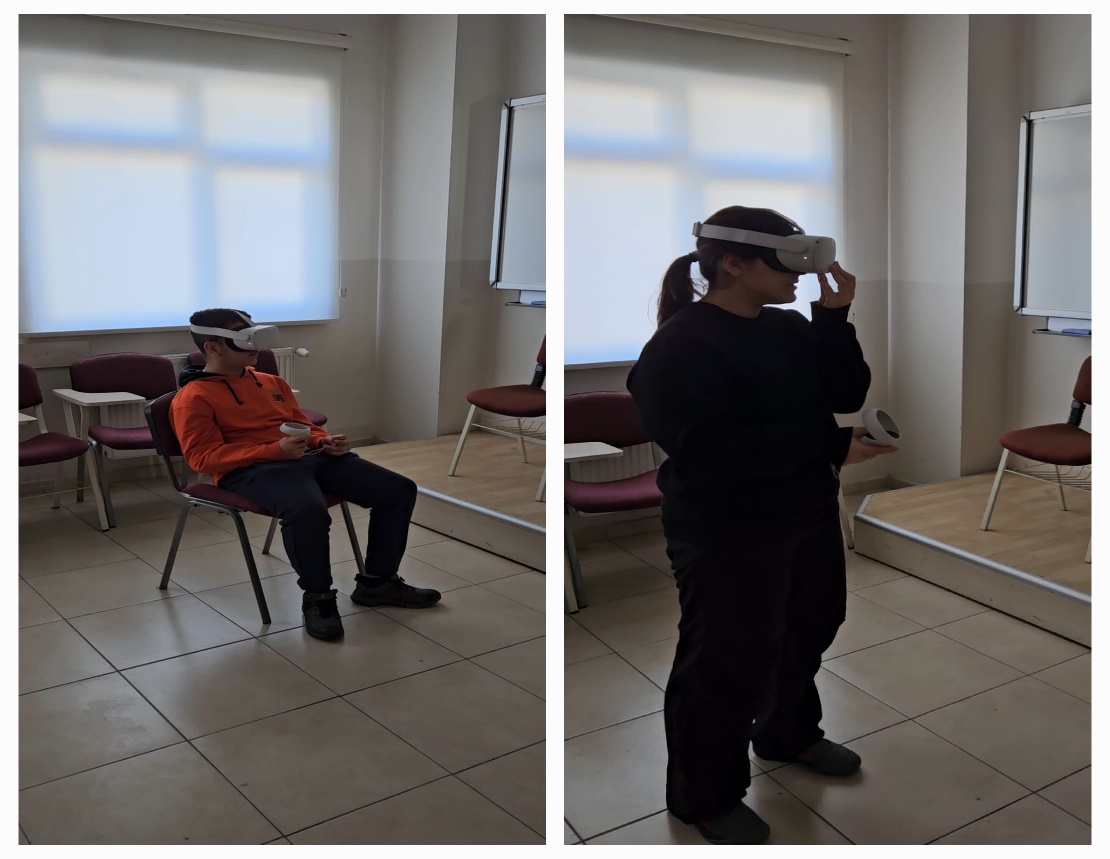


Appendix 5: **Ethics Committee Decision**


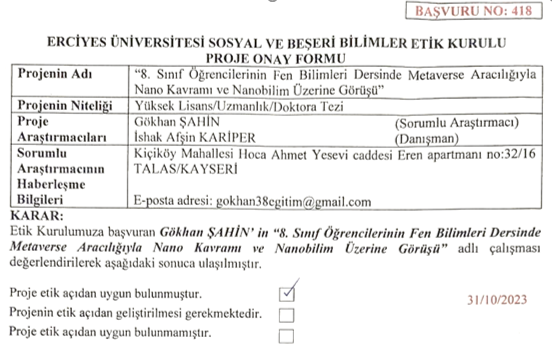


Appendix 6: **Student Responses, examples:**

To enhance transparency and illustrate the qualitative findings, representative excerpts from student responses obtained through interviews, observations, and document analysis are presented below. These examples are consistent with the themes reported in the Results section.

**A. Interview-Based Responses (Appendix 1)**

Question: What is nanotechnology?

K2 (before): “It is a kind of technology but I do not know exactly.”

D3 (after): “Nanotechnology is a technology that works at a very small scale like one billionth of a meter.”

Question: What is nanoscale?

K4 (before): “It is something very small.”

D2 (after): “Nanoscale means one billionth of a meter and it is smaller than atoms.”

Question: Where does nanoscale fit compared to atom and cell?

K1 (before): “I think it is small but I am not sure where it is.”

D5 (after): “Nano is smaller than atoms and much smaller than cells.”

Question: What do you think about learning with the Metaverse?

D1 (after): “It felt like I was inside a real laboratory.”

D4 (after): “It was like a game but I understood the lesson better.”

**B. Observation-Based Evidence (Appendix 2)**

During the Metaverse-based learning process:

- Students showed increased curiosity and engagement.

- Most students expressed excitement when using VR equipment.

- Students were more active and willing to participate compared to traditional instruction.

- The majority of students stated that they understood the topic better in the Metaverse environment.

- Students expressed a desire to continue similar lessons in future classes.

**C. Document Analysis Examples (Appendix 3)**

Pre-activity student drawing:

- Nano was represented as “a small dot” without scale awareness.

Post-activity student drawing:

- Nano was represented in relation to atoms and cells with a clear hierarchical structure (nano < atom < cell).

Pre-activity written response:

- “Nanotechnology is something small.”

Post-activity written response:

- “Nanotechnology is about structures at the nanoscale, and it is one billionth of a meter.”

These examples demonstrate the shift in students’ conceptual understanding, affective engagement, and ability to relate nanoscale concepts to prior knowledge, supporting the findings presented in the Results section.
